# Supplementary material for: PAS Domain-Containing Chemoreceptors Influence the Signal Sensing and Intestinal Colonization of Vibrio cholerae
Source: Genes (Basel). 2022 Nov 27;13(12):2224. doi: 10.3390/genes13122224 (PMC9777591; doi:10.3390/genes13122224)
Supplement: Supplementary file 1 [file genes-13-02224-s001.zip › genes-1954077-supplementary.pdf]

Supporting Information

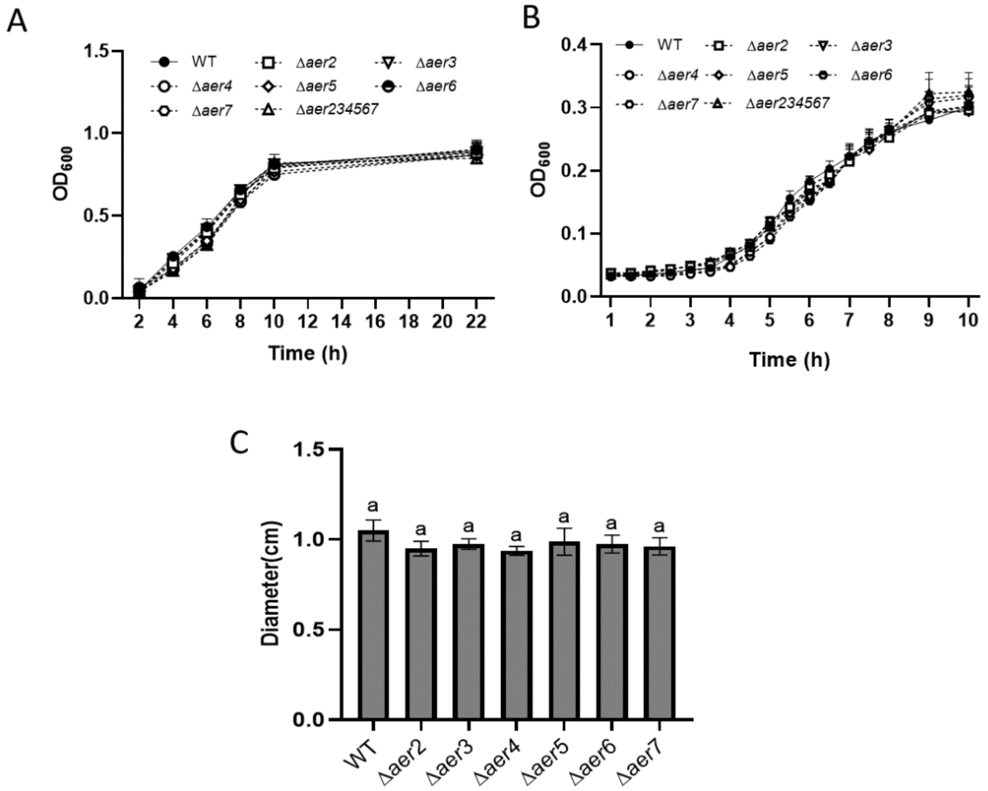

Figure S1. Growth curves of diverse *aer* mutants (A&B) and the detection of motility (C). Overnight cultures of the wildtype and *aer* mutants were inoculated 1:100 into LB (A) or M9 (B) broth. Then, the cultures were grown statically at 37°C, and at the time points indicated, the OD_{600} was measured. (C) Motility abilities of *aer* mutants. LB soft agar plates were incubated at 30°C for 24 h. Different letters in a single column indicate significant difference between treatments at the $p < 0.05$ level (One-way ANOVA).

Table S1. Strains and plasmids used in this study.

Strains or Plasmids	Relevant characteristics	Source
V. cholerae strains		
WT	*V. cholerae* C6706, wild-type, Sm^R	[1]
Δ*aer2*	Derivative of WT, VCA0658 in-frame deletion mutant, Sm^R	This study
Δ*aer3*	Derivative of WT, VCA0988 in-frame deletion mutant, Sm^R	This study
Δ*aer4*	Derivative of WT, VCA1092 in-frame deletion mutant, Sm^R	This study
Δ*aer5*	Derivative of WT, VC0098 in-frame deletion mutant, Sm^R	This study
Δ*aer6*	Derivative of WT, VC1406 in-frame deletion mutant, Sm^R	This study
Δ*aer7*	Derivative of WT, VCA0864 in-frame deletion mutant, Sm^R	This study
Δ*aer234567*	Derivative of WT, VCA0658, VCA0988, VCA1092, VC0988, VC1406, and VCA0864 in-frame deletion mutant, Sm^R	This study
WT (pSRKGm)	Derivative of WT carrying on vector pSRKGm, Sm^R, Gm^R	This study
Δ*aer2*^{C}	Derivative of Δ*aer2* harboring expression plasmid pSRKGm-*aer2*, Sm^R, Gm^R	This study
WT (P_{lac}-*aer2*)	Derivative of WT harboring expression plasmid pSRKGm-*aer2*, Sm^R, Gm^R	This study
WT (P_{lac}-*aer3*)	Derivative of WT harboring expression plasmid pSRKGm-*aer3*, Sm^R, Gm^R	This study
WT (P_{lac}-*aer4*)	Derivative of WT harboring expression plasmid pSRKGm-*aer4*, Sm^R, Gm^R	This study
WT (P_{lac}-*aer5*)	Derivative of WT harboring expression plasmid pSRKGm-*aer5*, Sm^R, Gm^R	This study
WT (P_{lac}-*aer6*)	Derivative of WT harboring expression plasmid pSRKGm-*aer6*, Sm^R, Gm^R	This study
WT (P_{lac}-*aer7*)	Derivative of WT harboring expression plasmid pSRKGm-*aer7*, Sm^R, Gm^R	This study
WT (*tcpP*-lux)	Derivative of WT harboring expression plasmid pBBRlux-*tcpP*, Sm^R, Cm^R	Lab strain
Δ*aer2* (*tcpP*-lux)	Derivative of Δ*aer2* harboring expression plasmid pBBRlux-*tcpP*, Sm^R, Cm^R	This study
Δ*aer234567* (*tcpP*-lux)	Derivative of Δ*aer234567* harboring expression plasmid pBBRlux-*tcpP*, Sm^R, Cm^R	This study
WT (*tcpA*-lux)	Derivative of WT harboring expression plasmid pBBRlux-*tcpA*, Sm^R, Cm^R	Lab strain
Δ*aer2* (*tcpA*-lux)	Derivative of Δ*aer2* harboring expression plasmid pBBRlux-*tcpA*, Sm^R, Cm^R	This study
Δ*aer234567* (*tcpA*-lux)	Derivative of Δ*aer234567* harboring expression plasmid pBBRlux-*tcpA*, Sm^R, Cm^R	This study
E. coli strains		
DH5α λpir	Host for cloning	[2]
SM10 λpir	Host for conjugation	[3]

Plasmids		
pWM91	Suicide cloning vector, Amp^R	[4]
pSRKGm	Gene expression vector for genetic complementation, Gm^R	[5]
pBBR-Lux	Transcriptional fusion vector, Cm^R	[6]

Table S2. PCR primers used in this study.

Primer	Sequence (5′-3′)[a]	Restriction sites

For deletion		
Δ*aer2*-1	CGGGATCCGTGTGATGACGAATCGAACTC	*Bam*H I
Δ*aer2*-2	TGCCCACGAGAGGGCATAGCCGATTCTG	
Δ*aer2*-3	GCTATGCCCTCTCGTGGGCAACACAAAC	
Δ*aer2*-4	AGGGGGCCCACTGACCGCATGTTTAACGAC	*Apa* I
Δ*aer3*-1	ATAAGAATGCGGCCGCGTGAAGCCGAAGCGGTGATC	*Not* I
Δ*aer3*-2	CTTTGTTTATACATTGCGCATAAAAGAGCTCC	
Δ*aer3*-3	TTTATGCGCAATGTATAAACAAAGCGCAAGAG	
Δ*aer3*-4	GGACTAGTATCGGCATGGTAAAGCCTTG	*Spe* I
Δ*aer4*-1	CGGGATCCACGTTGCGGCACAAGATAAG	*Bam*H I
Δ*aer4*-2	AAGACACATGGAATTCTAATCTAGGGATAACGTATG	
Δ*aer4*-3	ATTAGAATTCCATGTGTCTTCCCTCGTTAT	
Δ*aer4*-4	AGGGGGCCCAAAACGCCTGATGGGTTTAG	*Apa* I
Δ*aer5*-1	CGGGATCCTGACGCTTTACCCGATTACC	*Bam*H I
Δ*aer5*-2	AGAGCAGGTTTGAGCCTGGGTTGGATGAAA	
Δ*aer5*-3	CCCAGGCTCAAACCTGCTCTCCTTGGATG	
Δ*aer5*-4	AGGGGGCCCTCCGCGATTATCTCTGGAGC	*Apa* I
Δ*aer6*-1	CGGGATCCGCTACAGCTGCGATGTCTTG	*Bam*H I
Δ*aer6*-2	CCGGCTCATCAAATGGCTTCCAAATCAATGACTC	
Δ*aer6*-3	GAAGCCATTTGATGAGCCGGAAAGCCATCC	
Δ*aer6*-4	AGGGGGCCCTCGCGGAAAAAAGCAGAAAC	*Apa* I
Δ*aer7*-1	CGGGATCCACTCTGTAGTGAGCACAAAG	*Bam*H I
Δ*aer7*-2	GTTGGCTGACGTGAGGCATCGCTGAGATGG	
Δ*aer7*-3	GATGCCTCACGTCAGCCAACTCAAATAGTTGA	
Δ*aer7*-4	AGGGGGCCCGGCCGCTCGATAGATCATGC	*Apa* I
pWM91-F	TTCCCAGTCACGACGTTG	
pWM91-R	GCCAAGCGCGCAATTAAC	
For gene complementary or overexpression		
pSRKGm-*aer2*-F	GGGGTACCACTGAAATGGCCACATAACG	*Kpn* I
pSRKGm-*aer2*-R	GCTCTAGATGTGTTGCCCACGAGTC	*Xba* I
pSRKGm-*aer3*-F	GCTCTAGATATGCGCAATAACCAACCTG	*Xba* I
pSRKGm-*aer3*-R	GGGGTACCGTTTATACTCGGCGGAACTG	*Kpn* I
pSRKGm-*aer4*-F	GGGGTACCATTGACCGCCAACATACG	*Kpn* I
pSRKGm-*aer4*-R	GCTCTAGAACACATGGCATTTTGGAATCG	*Xba* I
pSRKGm-*aer5*-F	GGGGTACCACCCAGGCTCAAAACTCTC	*Kpn* I
pSRKGm-*aer5*-R	GCTCTAGAAGCAGGTTATGGGTTTATTGG	*Xba* I
pSRKGm-*aer6*-F	GCTCTAGAATTGATTTGGAAGCCATTTATGC	*Xba* I
pSRKGm-*aer6*-R	GGGGTACCTCCGGCTCATCTTATTCATC	*Kpn* I
pSRKGm-*aer7*-F	GCTCTAGACGATGCCTCACATTAAAGGG	*Xba* I
pSRKGm-*aer7*-R	GGGGTACCGAAGGGCAGTTTCTAGGATG	*Kpn* I
pSRK-F	ACGACCGGGTCGAATTTG	
pSRK-R	TCCGGCTCGTATGTTGTG	
For transcriptional fusion		
pBBRLux-F	TCATCGCAGTCGGCCTATTG	
pBBRLux-R	ATGCAACCGTAATTCGTTATTTC	

[a]The underline sequence is the restriction site of indicated enzymes.

References

1. Joelsson, A., Z. Liu, and J. Zhu, *Genetic and phenotypic diversity of quorum-sensing systems in clinical and environmental isolates of Vibrio cholerae.* Infect Immun, 2006. **74**(2): p. 1141-7.
2. Chart, H., et al., *An investigation into the pathogenic properties of Escherichia coli strains BLR, BL21, DH5alpha and EQ1.* J Appl Microbiol, 2000. **89**(6): p. 1048-58.
3. Ferrières, L., et al., *Silent mischief: bacteriophage Mu insertions contaminate products of Escherichia coli random mutagenesis performed using suicidal transposon delivery plasmids mobilized by broad-host-range RP4 conjugative machinery.* J Bacteriol, 2010. **192**(24): p. 6418-27.
4. WW, M., et al., *Conditionally replicative and conjugative plasmids carrying lacZ alpha for.* Plasmid. 1996 Jan;35(1):1-13. doi: 10.1006/plas.1996.0001., (0147-619X (Print)): p. 1-13.
5. SR, K., et al., *Broad-host-range expression vectors with tightly regulated promoters and their.* Appl Environ Microbiol. 2008 Aug;74(16):5053-62. doi: 10.1128/AEM.01098-08. Epub, (1098-5336 (Electronic)): p. 5053-62.
6. BK, H. and B. BL, *Regulatory small RNAs circumvent the conventional quorum sensing pathway in.* Proc Natl Acad Sci U S A. 2007 Jul 3;104(27):11145-9. doi:, (0027-8424 (Print)): p. 11145-9.
